# Can Text Messages Reach the Parts Other Process Measures Cannot Reach: An Evaluation of a Behavior Change Intervention Delivered by Mobile Phone?

**DOI:** 10.1371/journal.pone.0052621

**Published:** 2012-12-26

**Authors:** Linda Irvine, Donald W. Falconer, Claire Jones, Ian W. Ricketts, Brian Williams, Iain K. Crombie

**Affiliations:** 1 Centre for Biomedical Sciences and Public Health, University of Dundee, Dundee, Scotland, United Kingdom; 2 School of Computing, University of Dundee, Perth Road, Dundee, Scotland, United Kingdom; 3 The Nursing, Midwifery and Allied Health Professions Research Unit, University of Stirling, Scotland, United Kingdom; University of Pennsylvania, United States of America

## Abstract

**Background:**

Process evaluation is essential in developing, piloting and evaluating complex interventions. This often involves observation of intervention delivery and interviews with study participants. Mobile telephone interventions involve no face to face contact, making conventional process evaluation difficult. This study assesses the utility of novel techniques for process evaluation involving no face to face contact.

**Methods:**

Text messages were delivered to 34 disadvantaged men as part of a feasibility study of a brief alcohol intervention. Process evaluation focused on delivery of the text messages and responses received from study participants. The computerized delivery system captured data on receipt of the messages. The text messages, delivered over 28 days, included nine which asked questions. Responses to these questions served as one technique for process evaluation by ascertaining the nature of engagement with the study and with steps on the causal chain to behavior change.

**Results:**

A total of 646 SMS text messages were sent to participants. Of these, 613 messages (95%) were recorded as delivered to participants’ telephones. 88% of participants responded to messages that asked questions. There was little attenuation in responses to the questions across the intervention period. Content analysis of the responses revealed that participants engaged with text messages, thought deeply about their content and provided carefully considered personal responses to the questions.

**Conclusions:**

Socially disadvantaged men, a hard to reach population, engaged in a meaningful way over a sustained period with an interactive intervention delivered by text message. The novel process measures used in the study are unobtrusive, low cost and collect real-time data on all participants. They assessed the fidelity of delivery of the intervention and monitored retention in the study. They measured levels of engagement and identified participants’ reactions to components of the intervention. These methods provide a valuable addition to conventional process evaluation techniques.

## Introduction

Behavior change interventions delivered by text message to mobile telephones are becoming increasingly common [1]–[4]. The exceptionally high ownership of mobile telephones [5], and the popularity of text messaging as a preferred means of communication [6], ensures comprehensive access to target groups. The ability to deliver an intervention without face to face contact, not only provides an opportunity to reach large numbers of people at low cost, but may be particularly useful in recruiting people who are reluctant to engage in direct contact.

Process evaluation is essential in the development, piloting and evaluation of complex interventions [7]. It is used to assess the extent to which an intervention is deliverable in practice, in the way it was intended [7]–[10]. Conventional process evaluation often involves interviews, focus groups or observational studies, both with study participants and with those delivering the intervention [11]. A challenge for non-contact interventions is to measure process without introducing face to face contact during the intervention period, and thus altering the integrity of the intervention. This paper evaluates the utility of four novel methods of process evaluation that were developed for a brief alcohol intervention delivered by mobile telephone.

### Background to the Study being Evaluated

Alcohol-related morbidity and mortality present a major public health challenge [12], particularly among socially disadvantaged people [13], [14]. Tackling harmful drinking by disadvantaged men is a priority. Brief interventions are effective in reducing alcohol consumption [15], but they were designed to be delivered by health professionals in health care settings [15]. It is estimated that up to 85% of problems drinkers never access professional help [16]. The current approach to brief interventions therefore may not be sufficient to reach disadvantaged young to middle aged men who are seldom in contact with health services.

A brief intervention delivered by mobile phone was developed as an alternative method of reaching this high risk group. The intervention was tested in a feasibility study with men aged 25 to 44 years, who lived in areas of high social deprivation and had regular episodes of heavy drinking.

Two recruitment strategies were used: letters of invitation from GPs and respondent-driven sampling (RDS). All of the participants had mobile phones and regularly sent and received text messages. Two participants, whose phones were very outdated, were given new phones. Participants were required to pay for the texts messages which they sent to the researchers during the study. However, they were reimbursed by gift vouchers. Participants were given a £10 gift voucher (approximately UD$16) on completion of a baseline questionnaire. They were subsequently given one £5 voucher per week for the four week intervention period.

The intervention comprised a series of 36 Short Message Service (SMS) and Multimedia Messaging Service (MMS) messages which were sent to participants over a period of 28 days. The intervention was delivered by a computer system programmed to send the messages to the mobile phones of the participants in a predetermined sequence. The software was tailored to the specific requirements of this project. It combined the Application Programming Interface (API) provided by textlocal (www.textlocal.com) with PHP (scripting language), a MySQL database and server side scheduled tasks to control both text and media message delivery. Responses received from participants were stored electronically and analyzed on completion of the study.

Messages were constructed according to the conventions of texting, using the language of the target group. The content of the SMS and MMS messages was derived from several sources: alcohol brief interventions [15]; text message interventions [2], [3]; and communication theory [17]. The messages incorporated behavior change techniques using social cognition models [18] and motivational interviewing [19] and were organized by the stages of the Transtheoretical Model of behavior change [20]. The intervention guided participants through a series of steps towards reducing the frequency of binge drinking: recognition of reasons for drinking; awareness of alcohol-related harm and perceived benefits of cutting down; subjective norms and potential support from family and friends; control beliefs ie beliefs about factors that facilitate drinking less or impede drinking less; perceived behavioral control in changing drinking patterns; and intentions about future drinking. To promote interaction and to assess the impact of the components of the intervention, 9 of the 36 messages requested a response to a specific question. The responses to these messages formed an essential component for process evaluation. This paper reports on the utility of these process measures only.

Four techniques for process evaluation that did not involve face to face contact were used. Specifically, these measures were designed to assess: the fidelity of delivery of the intervention (the extent to which the text messages were delivered as intended); meaningful engagement with the messages (whether the messages were opened, read and responded to by participants); attenuation (whether engagement was sustained); engagement with the key components of the behavior change strategy; and ways in which the intervention could be improved.

## Materials and Methods

This feasibility study recruited 67 participants. Thirty-four men were randomised to the intervention group and 33 to the control group. As the detailed process evaluation described here was carried out on the intervention group only, this paper reports on the 34 men in the intervention group.

### Ethics Statement

The study was approved by the East of Scotland Research Ethics Service. Study participants provided informed consent by text message following a telephone discussion with the Research Fellow. All participants had previously received participant information leaflets by post. Participants were informed that some comments made by them could be included in papers on the study, but that all statements would be completely anonymous. All study data were de-identified and analysed anonymously. Data for the study were collected and analyzed between March and December 2011.

### Process Measure 1 Fidelity of the Delivery of the Intervention

The proportion of SMS messages delivered to participants was monitored as a measure of fidelity of the delivery of the intervention. SMS messages can be tracked to determine whether they were delivered as intended to the mobile telephone (it is not currently possible to track MMS messages). When SMS messages were not delivered to the telephone immediately, the computer program continued to try to send the message for 24 hours. If the message could not be delivered during this period, this was recorded as a delivery failure and the program would then send the next message in the sequence. Data captured on the delivery status of the SMS messages was recorded as: delivered (the phone had reception and was switched on); undelivered (the phone was switched off or it had no signal for 24 hours); or no status returned. The program could not record whether messages delivered to the phones were opened.

### Process Measure 2 Monitoring Initial and Sustained Engagement with the Study

Nine of the 36 text messages asked direct questions. Responses to the text messages were received and collated by the School of Computing at the University of Dundee. The anonymized messages were screened daily by a member of the research team who was not involved in recruiting the participants or delivering the intervention. Counting the number of responses provided a measure of engagement and retention in the study.

### Process Measure 3 Content Analysis of the Responses Received

Responses to the messages confirmed that the participants had opened and read the message; understood the question; reflected on the content/context of the question; and had given an appropriate and considered response. The content of the messages was analyzed to assess engagement with the study and with the psychological constructs of the behavior change strategy.

### Process Measure 4 Opportunities to Improve the Intervention

Text message responses to the questions were scrutinized to determine whether the messages had been interpreted as intended. This was used as a guide on how components of the intervention could be modified and improved.

## Results

### Characteristics of the Participants

The 34 participants were aged 25 to 44 years. More than three quarters (n = 26; 76%) of these men lived in the two most socially disadvantaged deciles (as measured by the Scottish Index of Multiple Deprivation [21]) and half (n = 17) had high school level qualifications only. Almost 60% (n = 20; 59%) of the participants were in employment and half of them (n = 17) lived with a partner.

The common pattern of alcohol consumption was one of occasional episodes of heavy drinking interspersed between periods of complete abstinence. More than one quarter of the men (n = 9; 26%) reported drinking more than 16 units in one session on more than five occasions in the previous month. Despite this, 27 men (79%) had more than 20 alcohol free days during that time. The Readiness to Change questionnaire [22], showed that most men (n = 24;71%) were in the pre-contemplation stage and few were taking action to change their behavior.

### Fidelity of the Delivery of the Intervention

The intervention package included 19 SMS messages which could be tracked electronically (the 17 MMS messages could not be tracked). A total of 646 SMS messages were sent to the 34 participants during the intervention period. Of these, 613 messages (95%) were recorded as delivered to the participants’ telephones. Of the remaining 33 messages, 28 were recorded as undelivered (the phone was switched off or it had no signal for 24 hours) and no delivery status was recorded for the remaining five messages. Six men had undelivered messages, (range 1 to 13, median 3.5). One man failed to receive six of the messages that asked a question; three men did not miss any of the questions; one missed two and one missed three questions. All but one of the men who failed to receive all of the text messages answered some of the questions.

### Frequency of Responses to Text Messages

**Figure 1:**
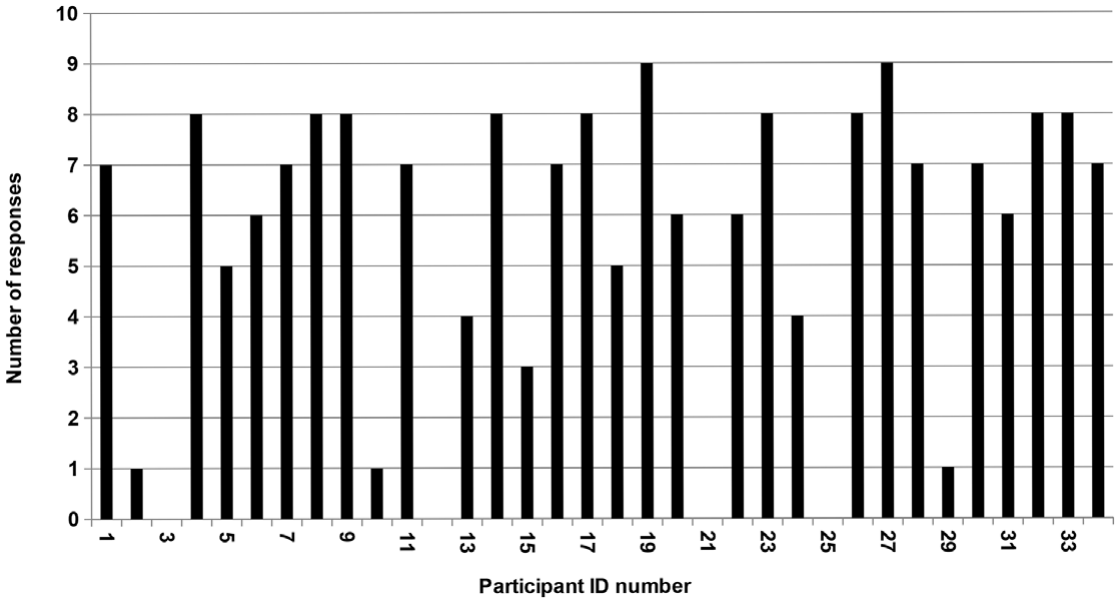
Participants’ text message responses to the nine questions. Nine of the 36 text messages sent to the study participants asked direct questions. Counting the number of responses provided a measure of engagement and retention in the study. Thirty participants (88%) responded to these questions. More than half (18 men; 53%) replied to seven or more of the nine questions with two replying to all nine questions.

Thirty participants (88%) responded to text messages that asked questions (Figure 1). More than half (n = 18; 53%) replied to seven or more of the nine questions with two replying to all nine questions and a further nine men answering eight questions. Four men did not respond to any of the questions and a further three only responded to one message.

**Figure 2:**
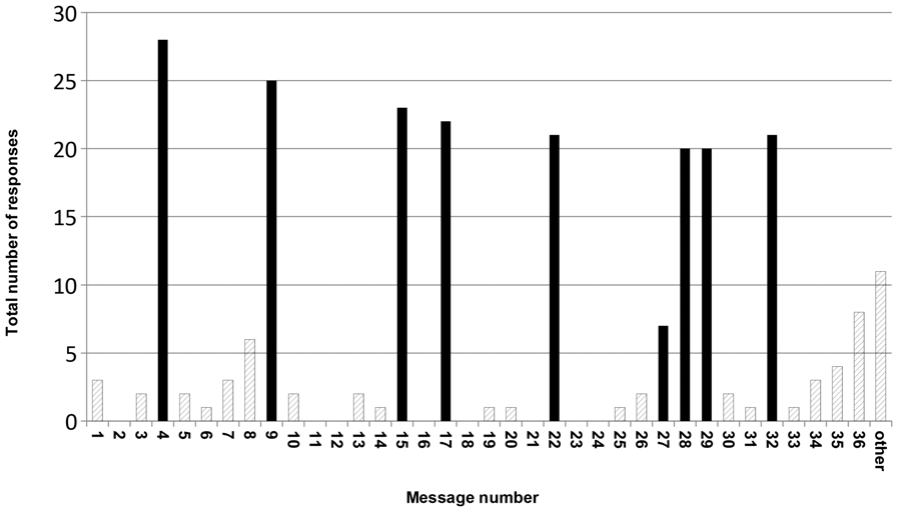
Total number of responses to text messages. Solid lines represent text messages that asked questions. More than 82% of men responded to the first question. There was little evidence of attenuation in responses across the intervention period. In addition to replying to the questions posed, many of the men responded spontaneously to other text messages (“other” category). Nineteen of the 27 messages which did not request a response received at least one reply.

More than 82% of men (n = 28) responded to the first question (Figure 2). For the remaining eight questions, an average of 20 men responded. Overall, there was little evidence of attenuation in responses across the intervention period. In addition to replying to the questions posed, many of the men responded spontaneously to other text messages. Nineteen of the 27 messages which did not request a response received at least one reply (Figure 2). Some responses simply acknowledged that the message had been delivered, expressed empathy with the message, or responded to the humor in the message and some were thank you messages from participants at the end of the study.

### Content of the Responses Received

Responses to the questions demonstrated engagement with cognitive antecedents to reduced drinking. Most were lengthy for text messages and appeared to have been well thought out. Illustrative responses are presented by the intended impact of the text message questions.

#### Recognition of reasons for drinking

An early message was designed to identify the type of drinkers in the study. Based on the Drinking Motives Questionnaire (DMQ) [23], which categorizes reasons for drinking as social, coping or enhancement, the message asked: “What’s the main reason U drink? A. It’s a habit; B. To feel better; C. To have fun; D. To cope. Text me your answer”. The majority of men (23 of 34) indicated that they drink *“to have fun”*, although some gave more than one answer. Four men said that their drinking was *“a habit”*, while another five reported that they drink *“to feel better”*. Only one man said that he drank *“to cope”*. One man gave a detailed reason for drinking: *“To have fun socialise and let my hair down. I work hard all week and when I get the chance I feel I deserve to enjoy my weekend”.* The responses confirmed that the participants largely considered themselves to be social drinkers.

#### Awareness of the harms of heavy drinking/Perceived benefits of drinking less

“Can U think of any reasons why it may be a good idea for U to cut down a bit on your drinking? Text me your answer!” was designed to encourage re-evaluation of current drinking behavior. It was posed as a question so that the participant would not only voice an argument for change, but write it down in a text and return it to the researcher. Although the men saw themselves as social drinkers, they acknowledged the negative effects, and were able to identify benefits of reduced drinking, both in the immediate future, and importantly in the long term. Responses to the question fell into four categories:

Immediate benefits.

*“I wouldn't feel like crap in the morning, and my wallet would have more money in it!”*

*“I would want to cut down drinking to enjoy my night more and not forget parts of it. it would save on cash and avoid sore heads in the morning”*

*“feelin rough hangovers getin into silly situations getin into trouble all these things get u at some point with 2 much drinkin!”*

Health benefits.

*“Unhealthy and bad liver”*

*“To stay healthier later in life.”*

Family reasons.

*“To get fit and stay healthy for my family”*

*“Live longer for my kids”*

Financial benefits.

*“Money”*

*“Save money no get hungover.”*

Some text messages were delivered in pairs as a means of reinforcing important concepts, and to extend the narrative of the message. Two messages, delivered in quick succession, were designed to encourage the participants to think about the pros and cons of changing their drinking patterns. The first simply asked “How much would U save every month if U drank half as much?” The second, delivered three minutes later, said “Please count up your savings & text me the sum!” Instead of seeing this as an intrusion of their privacy, the men gave it careful consideration. More than 70% did the calculation and responded, revealing that their estimated savings ranged from £10 to £690 (approximately US $16– $1,100).

*“Hard 2 tell. But i would have at least saved 24 pound so far this week.”*

*“About between fifty and eighty pounds and thats a fact”*

*“£200 a month or more easy. That would be on carry outs and the pub.”*

The next two text messages developed this theme by asking how saving money could enable them to buy items they wanted. This transforms potential benefits from the abstract to concrete. The first message stated “By saving up your cash U could treat yourself to something special!” The second, delivered three minutes later said “Try to picture what U would like to buy & text me back your answer!” The men identified a range of ways to spend the saved money from simple treats to extravagant holidays. Responses demonstrated that the men had thought seriously about the potential opportunities offered by having extra money:

*“saving that money would help me take my girlfriend out for a meal now and then”*

*“Definitely a car possibly a few more holidays - love buying designer clothes”*

*“Trek 2.5 road bike - cost £1650.00 RR”*

*“Holiday to Australia for 3 weeks.”*

One message used a quotation from a former heavy drinker: “Andy from Dundee says – “I cut back on my drinking because my father in law died of it” What would be a good reason for U to cut back? Text me back!” This elicited deeply personal responses, again both on the short term and long term benefits:

*“I really wanna stay out of trouble and not become the person I can be after a few too many”*

*“I would b able to make the most of the next day rather than feeling shite”*

*“Ive have tryed because i seen my dad nearly die”*

*“Good reason for me cutting back was again, looking after my son. I can't allow drinking to interfere with my job either. My Grandad was an alcoholic, so I know the health risks associated with booze.”*

#### Subjective norms

“Can U think of someone who’d be happy if you made a change! What would U hear them say? Please text me your answer?” This text message prompted participants to identify people who would approve of their decision to reduce their alcohol consumption. Parents, partners, family members and friends were identified as people who would be pleased to see a reduction in drinking. Some men gave detailed responses on what their family and friends would say:

*“Thats brilliant what u have done,maybe we can do something at the weekend”*

*“yes my friends & family would say well done & good on you keep it up & stay focused & positive abou life because u only get 1 chance.”*

Two of the men gave a light hearted but nevertheless thoughtful response:

*“they would say what a peaceful night not having to deal with a drunken ass”*

*“My dad. Its good ur no phoning me for a lift at 2am!”*

#### Control beliefs/Perceived behavioral control

The final question in the intervention package was a multiple choice question which was intended to both motivate and challenge the participants. It was also designed to encourage the belief that change is possible. The message stated “Many people find it easy to reduce their drinking. Do U think U could if U tried? A Yes; B No; C Maybe. Please text me your answer!” This question was given an overwhelming positive response by the men who responded. Seventeen of the 21 who responded answered yes, with one man saying *“A, for sure*”. Four men said “maybe” and none said “no”.

### Behavioral Intentions

One of the last messages stated “Liver disease (cirrhosis) is the major cause of death in heavy drinkers. Drinking less will greatly reduce your risk of liver failure.” This message was not phrased as a question and did not seek a response. However, one spontaneous response to this message was: *“Im away 2 try and cut down or stop from monday.”* This indicates that text messages can motivate intention to change, a key step in behavior change.

### Identifying Ways to Improve the Intervention

#### Ambiguous question

One question, “Can U think of any obstacles or barriers that stop U drinking a bit less each week? Text me your answer!”, was misinterpreted by approximately half of the men who responded. While the question asked for barriers to reducing alcohol consumption, some men listed factors which facilitated drinking less:

*“Work goin someplace in the car. Kids commitments.”*

*“Driving and work and playing football and definitely when I look after my daughter!”*

*“Prices going up.”*

*“Yea money, getting up early for work with hangover, and prices in pubs, wow”*

Some men, however, were able to identify barriers.

*“I dont always know when iv had enough”*

*“My friends asking me to meet them in the pub for a couple”*

*“Boredom habit stress”*

The question was effective in encouraging the men to identify barriers, so it should be retained, but re-phrased to be more easily understood.

#### Unpopular question

One question proved unpopular, with only seven men answering it. It asked “How much did U spend on alcohol this week – Please text me your answer!” The message was designed to make the participants think about the pros and cons of drinking, and to highlight the negative consequences. The amount spent ranged from zero to:

*“55 quid I reckon which isn't bad! I think;-)”*

*“Ive spent seventy six pounds this week ok mate”*

Those who responded gave appropriate answers, so it is unlikely that the question was misunderstood. However, the men had already answered questions that addressed how much money they could save if they drank less. The low response suggests that participants felt the topic had already been covered. Most studies repeat messages given in an intervention as a means of reinforcing the intervention [2]. This message contained one of the few loss framed questions [24], and indicates that this approach should be avoided.

## Discussion

This study has demonstrated the value of process evaluation by monitoring the delivery of text messages and assessing the responses to text message questions. In particular it provided a method of assessing the extent of participants’ engagement with the intervention. Text message questions have been used in previous trials to promote interactivity [2], [3], but have not been used to monitor process. This non-contact approach found that the client group (disadvantaged young and middle aged men) are very willing to engage in a study with an interactive intervention delivered by mobile phone. It also showed that participants had not only received, opened and read the messages, but thought deeply about the content and had taken the time to respond. Many of the men gave carefully considered personal responses to the questions set. Interest in the intervention was maintained for the duration of the study period with very little attenuation in the number of men responding to text messages.

The four novel methods described were successful in fulfilling many of the requirements of process evaluation. They established that the components of the intervention were delivered consistently and accurately to the target group [8], [25]. They monitored engagement with the intervention; and the likely uptake and engagement of the intervention with the target population [11]. Comprehension of the messages was monitored by assessing the nature of responses to the questions. Bellg et al state that measuring fidelity of delivery of the intervention should include participants’ understanding of the information given, particularly if literacy levels are low [26]. The methods used also helped to identify what factors could have contributed to the high engagement e.g. humor and empathy; and how further improvements could be made to the design of the intervention e.g. avoid repetition of topics and loss framed messages [7], [27]. Finally, the approach enabled monitoring of the retention of participants in the study [28]–[31].

Engagement with components of the behavior change intervention was assessed by analyzing the text messages received from participants. Responses to the questions indicated engagement with cognitive antecedents to reducing drinking, and with important steps on the causal chain to behavior change. A notable feature of the responses to text questions is that there was a high level of engagement from the start of the study with little sign of attenuation during the course of the intervention. The target group are frequent mobile phone users, and were apparently happy to engage in conversations. This could explain why there was little attenuation in the responses. Mobile phone etiquette requires reciprocation, so that messages from the person who initiates the exchange are likely to be answered [32]. It is conventional for text message conversations to involve several exchanges. The source of the messages (University of Dundee) was seen to be credible, which is also an important factor in engagement [33]. Another early indication that the men were willing to engage in a narrative was in responses to multiple choice questions. Very few men gave the one letter multiple choice answer: instead they transcribed the whole answer e.g. in response to the question on reasons for drinking a typical reply was *“C - to have fun”*. The high response indicates that men were comfortable with the study.

A key finding is the value of content analysis of the responses to the questions asked. This provides a further important dimension of fidelity that has not been previously reported [2]. Obtaining text message replies to carefully crafted questions provides an ideal mechanism for examining the impact of components of a behavior change intervention.

There are several other advantages of using this methodology. Critically, the process evaluation did not alter the intervention. The intervention was designed to have no face to face contact, so that if found to be effective, it could be rolled out to large numbers of people at low cost. Obtaining process data during the intervention period through interview or focus groups would have altered the integrity of the non-contact method.

Conventional process evaluation is usually conducted with a subgroup of the study population. The process measures used in this study collected data on all participants. Thus, issues around sampling and the representativeness of those included were eliminated. A further benefit is that the costs of data collection are low.

Another attraction of this method is that it is unobtrusive. Questions that explored engagement with components of the behavior change intervention were embedded in a series of non-threatening text messages. Participants could choose to ignore these questions, with no likelihood of being pressed for an answer. The result was that personal questions were asked in a way which elicited deeply personal and apparently honest answers. An added advantage is that the questions were answered in ‘real time’ and in the ‘real world’ [16]. Thus the responses could monitor the impact of components of the intervention on participants as they were going about their daily business. This method of process evaluation also avoids problems with recall and rationalization that may occur at a post study evaluation. The success of this method suggests that text messaging could be used in a range of trials, not only those where the intervention is delivered by mobile phone.

One major limitation of this approach is that currently MMS messages cannot be tracked. Thus, the results are based on the delivery of SMS messages only, which accounted for 53% of the total messages in the intervention package. Failure to receive messages is a potential problem although in this study it was found to have minimal impact. Another limitation of this study was the low number of participants as it was a feasibility study only. However, the numbers were sufficient to confirm that the process measures used are suitable and effective for electronic interventions.

### Conclusion

This feasibility study has identified an effective means for a detailed process evaluation of a complex intervention delivered electronically. The method offers many advantages. It collects real time data, unobtrusively, from all participants. Content analysis of responses to text messages confirmed fidelity of the delivery of the intervention. Crucially, it also measured the extent of engagement with components of the behavior change strategy, identified ambiguity in messages, highlighted gaps in the intervention and areas for improvement. Used sensitively the techniques described will identify interventions which are likely to fail and will highlight components of the intervention that need modification. These novel methods provide a valuable addition to conventional techniques for process evaluation.
